# Hyperparathyroidism in Pregnancy Leading to Pancreatitis and Preeclampsia with Severe Features

**DOI:** 10.1155/2017/6061313

**Published:** 2017-04-11

**Authors:** Andrew G. Dale, Bradley D. Holbrook, Lauren Sobel, Valerie J. Rappaport

**Affiliations:** ^1^Department of Obstetrics & Gynecology, University of New Mexico, Albuquerque, NM, USA; ^2^Touro University College of Osteopathic Medicine, Vallejo, CA, USA

## Abstract

*Background*. Hyperparathyroidism is underdiagnosed in pregnancy, yet early diagnosis is necessary for the potentially severe sequelae of hypercalcemia for both the woman and fetus.* Case*. A 31-year-old, gravida 3, para 0-0-2-0 at 32 weeks and 3 days of gestation, presented with preeclampsia with severe features concomitant with acute pancreatitis and known diabetes mellitus type 2. She was stabilized and delivered. In the postpartum period, her total calcium level remained elevated. Ionized calcium levels and parathyroid hormone levels were also elevated, and she was diagnosed with hyperparathyroidism.* Conclusion*. Hyperparathyroidism and hypercalcemia are risk factors for pancreatitis. Women who develop pancreatitis during pregnancy are at increased risk of developing preeclampsia. If elevated serum calcium is noted, it should be confirmed with ionized calcium level and parathyroid hormones as ionized calcium levels are unaffected by pregnancy.

## 1. Introduction

Primary hyperparathyroidism (PHP) is a disorder of calcium homeostasis caused by inappropriate overproduction of parathyroid hormone. It is the third most common endocrinologic disorder, with a prevalence estimated at 1.4% [1, 2]. It primarily affects women, with a female-to-male ratio of 3 : 1 [3]. The incidence of PHP during pregnancy is not known.

Hyperparathyroidism can lead to a variety of clinical symptoms, including nephrolithiasis, depression, constipation, bone fracture, maternal cardiac dysrhythmia, and hyperemesis gravidarum. The diagnosis of primary hyperparathyroidism is usually performed with an initial series of lab tests involving serum calcium levels including an ionized calcium, direct measuring of parathyroid hormone, or sestamibi radiouptake scans. Sestamibi radiouptake scans are useful for localizing the lesion but are not considered diagnostic. Unfortunately, in a pregnant patient population, standard laboratory tests can be misleading, as was seen in our patient's initial standard work-up, and nuclear studies are contraindicated. Diagnosis can be further complicated by the masking of clinical symptoms due to the similarity to common complaints of pregnancy.

Acute pancreatitis is also a rare finding in pregnancy, with an incidence ranging from 0.02 to 0.1% [4]. While rare in pregnancy, it has the potential to cause significant morbidity and mortality to both mother and fetus. The most common cause of pancreatitis in pregnancy is biliary obstruction from gallstones. Acute pancreatitis during pregnancy has also been associated with preeclampsia [5] as well as hypercalcemia [6].

## 2. Case

A 31-year-old gravida 3, para 0-0-2-0 at 32 weeks and 3 days, was transported to our hospital from an outside facility due to concern for preeclampsia with severe features based on severely elevated blood pressures accompanied by scotomata and epigastric pain. Her pregnancy had also been complicated by type 2 diabetes mellitus, which had been poorly controlled on insulin and metformin. On admission, her hemoglobin A1c was 8.7%. Standard preeclampsia labs, AST, ALT, uric acid, creatinine, complete blood counts, and lactate dehydrogenase, as well as acute abdominal pain labs, lipase, amylase, liver function tests, and chemistries, were ordered. Labs revealed a mild leukocytosis at 14.7 × 10^3^/uL but blood counts, chemistry, and liver function tests were otherwise within normal limits with calcium at 9.9 mg/dL (ref range 8.4–10.4 mg/dL).

The patient was started on IV labetalol for blood pressure control and magnesium sulfate for seizure prophylaxis. She received a course of antenatal corticosteroids. Despite her normal labs and improvement of her blood pressures and scotomata, her midepigastric pain persisted. Abdominal imaging revealed no abnormalities, but a lipase was found to be severely elevated at 7235 unit/L (reference range 66–360 unit/L) and an amylase of 754 unit/L (reference range 21–116 unit/L).

Her pancreatitis was managed with supportive care including IV fluids and bowel rest. Her epigastric pain did not resolve, and for this reason the decision was made to proceed with delivery. She underwent a primary cesarean delivery due to first-stage arrest of labor and delivered a healthy infant weighing 2400 g, with APGARs of 8 and 9 at 1 and 5 minutes, respectively. On postoperative day two, it was noted that her calcium was near the upper limits of normal at 10.2 mg/dL. An ionized calcium level confirmed the presence of hypercalcemia, with an elevated value of 1.58 mmol/L (ref range 1.15–1.27 mmol/L). We then checked her parathyroid hormone level, which was also elevated at 122 pg/mL (ref range 11–88 pg/mL). A neck ultrasound revealed an ovoid mass measuring 1.7 × 0.8 × 0.9 cm, with increased blood flow, findings consistent with a likely parathyroid adenoma.

The patient's pancreatitis slowly improved and she was discharged home on postoperative day number 5 in stable condition. At her postpartum follow-up visit, it was noted that her insulin demands were increased above the dosage that was required prior to her prolonged course of pancreatitis. All other vital signs were within normal limits. A referral to endocrinology was placed to follow labs associated with the presumed hyperparathyroid adenoma. She is also being followed in the otolaryngology clinic for possible surgical resection of her parathyroid adenoma. Later chart review showed that she went on to have resection of this with pathology consistent with parathyroid adenoma.

## 3. Discussion

PHP and resultant hypercalcemia can have significant consequences to a gravid woman and her developing fetus. Hypercalcemia has a wide spectrum of presentations depending on the degree of serum elevation. Mild hypercalcemia, defined as less than 12 mg/dL, produces vague symptoms which are similar to common complaints of pregnancy such as fatigue, constipation, nausea, vomiting, and depression. However, once hypercalcemia becomes more severe, with levels above 13.0 mg/dL, the resultant symptoms are much less commonly seen in normal pregnancy. These include renal impairment, mental status changes, cardiac arrhythmias, renal calculi, osteopenia, peptic ulcer disease, pseudogout, and muscle atrophy. Profound hypercalcemia, with levels greater than 14-15 mg/dL, should be considered as a medical emergency and treated as such [7].

In addition to affecting maternal health, maternal hypercalcemia can directly affect the fetus as well. Calcium is actively transported across the placenta and fetal serum calcium levels are maintained at a higher level than that of a mother. There is evidence that maternal hypercalcemia leads to an increase in net placental calcium transport, which can excessively suppress the fetal parathyroid glands [8]. Suppression of the fetal parathyroid may lead to neonatal hypocalcemia, and in rare cases, this hypocalcemia may persist and become permanent. Permanent hypocalcemia is thought to be due to teratogenic effects of hypercalcemia on the third and fourth brachial clefts [9]. Identification of neonatal hypocalcemia is extremely important in that if not corrected it can lead to tetany with respiratory failure in the first few hours of life.

The diagnosis of PHP can be a challenge in pregnancy since elevated serum calcium, one of the primary features of this disease, may not be diagnostic. Total serum calcium levels are lower in pregnancy due to physiologic changes including decreased albumin levels, facilitated transport of calcium across the placenta, and increased glomerular filtration rate [7]. Therefore, an ionized calcium level should be the first step for the initial work-up when PHP is suspected. These levels are fortunately not affected by pregnancy.

Pancreatitis itself is also rare and morbid medical condition in pregnancy. Recent studies show that pancreatitis in pregnancy carries maternal and fetal mortality rates of <1% and <5%, respectively [5]. Aside from mortality, there are several other documented maternal and fetal/neonatal complications associated with pancreatitis in pregnancy. These include preeclampsia (odds ratio 4.21), preeclampsia with severe features (OR 7.85), preterm delivery at less than 32 weeks (OR 3.31) and 37 weeks (OR 4.10), neonatal respiratory distress syndrome (OR 4.27), and intrauterine fetal demise (OR 4.35) [5].

Risk factors for the development of acute pancreatitis during pregnancy include gallstones, hyperlipidemia, alcohol consumption, and genetic variants [5, 10]. Our patient had another rare cause of pancreatitis, due to hyperparathyroid-induced hypercalcemia.

Hypercalcemia is also a recognized risk factor in the development of acute pancreatitis; it is estimated to account for 1.5–7.0% of cases of acute pancreatitis in the general population [11]. The presentation of acute pancreatitis secondary to hypercalcemia in the setting of PHP commonly includes the symptoms of vomiting and abdominal pain, as is commonly seen with pancreatitis of any etiology. However, in the setting of pancreatitis secondary to severe hypercalcemia, transient seizure, eye deviation, and impaired visual fields may also be seen [4]. These severe findings are significant in that their presence should prompt providers to pursue ionized calcium levels.

In summary, PHP is likely underdiagnosed in pregnancy, but prompt diagnosis is important. Ionized calcium is the initial diagnostic test for this disease and if elevated should be followed by parathyroid hormone levels and potentially imaging studies. Undiagnosed PHP and associated hypercalcemia can lead to significant complications for both mother and fetus, including preeclampsia, pancreatitis, and intrauterine fetal demise. In addition, the long-term health effects for the patient may be significant if the diagnosis is missed or delayed. As such, early diagnosis and treatment of this disease are critical to prevent these potentially profound clinical sequelae.

## Additional Points

*Teaching Points*. (1) Hyperparathyroidism is the third most common endocrinologic disorder and should be considered if symptoms of hypercalcemia are present. (2) Hypercalcemia can lead to pancreatitis. (3) Ionized calcium levels are not altered during pregnancy and should be the initial test in the work-up of suspected hyperparathyroidism.
